# Seasonal variation in the association between household food insecurity and child undernutrition in Bangladesh: Mediating role of child dietary diversity

**DOI:** 10.1111/mcn.13465

**Published:** 2022-12-07

**Authors:** Md. Mehedi Hasan, Abdul Kader, Chowdhury Abdullah Al Asif, Aminuzzaman Talukder

**Affiliations:** ^1^ Helen Keller Intl Country Office Dhaka Bangladesh

**Keywords:** Bangladesh, child dietary diversity, food insecurity, nutrition, seasonality

## Abstract

Household food insecurity (HFI) and child dietary diversity (CDD) are variable across seasons. We examined seasonal variation in HFI and child undernutrition association and tested how CDD mediates this association. We analyzed data for 26,353 children aged 6–59 months drawn from nationally representative cross‐sectional Food Security and Nutrition Surveillance Project data collected during 2012–2014 in Bangladesh across three seasons annually: Post‐Aman harvest (January–April); Monsoon (May–August); and Post‐Aus harvest (September–December). Multivariable logistic regression analysis adjusted for individual, maternal, household and geographical characteristics reveals that children of food‐insecure households were more likely than food‐secure households to be stunted (adjusted odds ratio, AOR: 1.12; 95% confidence interval, CI: 1.02–1.23; *p* < 0.05), wasted (AOR: 1.21; 95% CI: 1.05–1.39; *p* < 0.01) and underweight (AOR: 1.16; 95% CI: 1.04–1.3; *p* < 0.01). CDD mediated 6.1% of the total effect of HFI on underweight. These findings varied across seasons. HFI was associated with greater odds of underweight during Monsoon (AOR: 1.32; 95% CI: 1.08–1.62; *p* < 0.01) and Post‐Aus (AOR: 1.21; 95% CI: 1.06–1.37; *p* < 0.01) while wasting during Post‐Aus (AOR: 1.65; 95% CI: 1.35–2.01; *p* < 0.001). CDD largely mediated the total effect of HFI on underweight during the Post‐Aman in 2012–2014 (23.2%). CDD largely mediated the total effect of HFI on wasting (39.7%) during Post‐Aman season in 2014 and on underweight (13.7%) during the same season in 2012. These findings demonstrate that HFI is seasonally associated with child undernutrition and mediated by CDD as well in Bangladesh and seasonality and diversity should be considered while designing appropriate population‐level food‐based interventions to resolve child undernutrition.

## Key messages

- The prevalence of child undernutrition was greater among children of food‐insecure households than food‐secure counterparts.
- Children of food‐insecure households were more likely than food‐secure households to be stunted, wasted and underweight.
- Child dietary diversity mediated the relationship between food insecurity and child undernutrition, but this mediation effect fluctuated across seasons.

## Graphical abstract

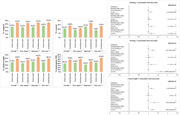

## 1 INTRODUCTION

Child undernutrition continues as a global burden, accounting for an estimated 149 million stunted children under 5 years of age and 45 million wasted in 2020 worldwide (World Health Organization [WHO], 2021). Bangladesh made substantial progress in reducing child undernutrition over the last two decades, with the rate of stunting dropping from 60% to 28%, wasting by more than half (from 21% to 10%) and underweight by 30 percentage points (from 52% to 22%) from 1996–1997 to 2017–2018 (National Institute of Population Research and Training [NIPORT] and ICF, 2020). Despite this exemplary reduction, child undernutrition is still a very high concern for stunting (≥30%) (WHO, 2019) and underweight (≥30%) (WHO, 2010b), and high concern for wasting (10%–<15%) (WHO, 2019).

Considering the high burden of child undernutrition and its short‐ and long‐term consequences, it is imperative to understand the role of key factors that drive this burden. The factors that affect child undernutrition are multifaceted and include both underlying (e.g., adequate nutrition, education, food, dietary practices) and immediate reasons (e.g., morbidity). Household food insecurity (HFI) constitutes one of the underlying factors of child undernutrition according to UNICEF's well‐established conceptual framework for undernutrition (UNICEF, 1998). However, the literature shows a mixed association between HFI and child undernutrition. While a positive association between HFI and child undernutrition is common in many settings such as Colombia (Hackett et al., 2009) and the United States (Matheson et al., 2002) including Bangladesh (Hasan et al., 2013; Saha et al., 2009), no significant association between these two components has also been found (Alaimo et al., 2001; Bhattacharya et al., 2004; Kaiser et al., 2002; Osei et al., 2010). Further, HFI has been found to affect child dietary diversity (CDD), a potential direct and/or indirect factor for child undernutrition (Chandrasekhar et al., 2017). The magnitude of HFI can be modifiable and could vary from context to context or from season to season within the same context (Raihan et al., 2018). Given the effect of seasonality on HFI, consumption of diversified diets and undernutrition of children is likely to be influenced by seasonality as well.

While several studies have investigated the association between season and HFI (Raihan et al., 2018), there are no prior studies that have established the mechanism of how seasonal variation leads to changes in HFI and child undernutrition association. Also, less is known about the mechanism of how CDD works in this pathway across different seasons. This study investigates the association between HFI and child undernutrition across seasons and the role of CDD in this pathway to better understand the variations in this mechanism to design and deliver season‐specific policies and programs.

It is hypothesized that HFI varies across seasons and results in variations in CDD and undernutrition rates. In this study, we examined the seasonal variation in the association between HFI and child undernutrition and tested the mediating role of CDD in this pathway.

## 2 METHODS

### 2.1 Data

We used nationally representative repeated cross‐sectional Food Security and Nutritional Surveillance Project (FSNSP) data collected during 2012–2014. The FSNSP collected data every year to track variations of a range of indicators including but not limited to nutrition and food security over the three seasons in Bangladesh. Based on the harvest period of three major types of rice in Bangladesh, the FSNSP defined these three seasons as follows: Post‐Aman harvest season (January–April); the height of the Monsoon (May–August), which is typically the Boro harvest season in Bangladesh; and the Post‐Aus harvest season (September–December) (Helen Keller International [HKI] & BRAC Institute of Global Health [BIGH], 2013; HKI & James P. Grant School of Public Health [JPGSPH], 2016; HKI & JPGSPH, 2014). In Bangladesh, harvest periods create employment opportunities to generate income, especially for those who live below the poverty line. Employment and resulted income generation opportunities are typically reduced during the post‐harvest seasons (Post‐Aman or Post‐Aus). In Bangladesh, these seasons are known as lean seasons due to the lack of employment opportunities and reduced income generation (Raihan et al., 2018).

The FSNSP implemented a multistage sampling design by identifying vulnerable zones for targeted surveillance to collect a nationwide sample. In the first stage, the FSNSP divided the country into 13 strata, which corresponded to the six surveillance zones (coastal belt, eastern hills, haor region, padma chars, northern chars and the northwest region) and administrative divisions (Dhaka, Chittagong, Rajshahi, Barisal, Khulna, Sylhet and Rangpur). From each agroecological zone, 12 upazilas (subdistrict) were selected with replacement by rotation, while 22 upazilas were selected with replacement but without rotation (stratified by division) from the rest of the country. All villages/mohallas under each upazila were considered as a community (cluster of households). The FSNSP randomly chose four communities from all the communities in each upazila at the second stage and 24 households from each selected community at the third stage of sampling. The FSNSP selected and surveyed households with at least one woman 10–49 years of age or a child less than 5 years of age. A detailed description of the FSNSP methodology can be found in the final FSNSP reports (HKI & BIGH, 2013; HKI & JPGSPH, 2014, 2016).

During 2012–2014, the FSNSP surveyed 77,036 households (27,068 households in 2012, 22,896 households in 2013 and 27,072 households in 2014). Information on childhood characteristics including anthropometry was collected for 29,453 children aged 0–59 months (10,092 children in 2012, 8870 children in 2013 and 10,491 children in 2014). Although the FSNSP‐measured anthropometry for all eligible children 0–59 months of age lived in the selected households, information on dietary consumption was collected only for the youngest child 6–59 months of age. Therefore, we restricted our analysis to this cohort to assess the role of CDD in HFI and undernutrition association. In the final analysis, we analyzed 26,353 households/mother–child (youngest children aged 6–59 months) dyads (8969 children in 2012, 8030 children in 2013 and 9354 children in 2014) and excluded 313 cases due to flagged/missing values (see Figure S1 for detailed sample selection procedure). For this paper, we pooled the FSNSP data collected in 2012, 2013 and 2014 to understand pooled estimates.

### 2.2 Outcome variables

The outcome variables of this study were stunting (low height‐for‐age), wasting (low weight‐for‐height) and underweight (low weight‐for‐age). The FSNSP measured the weight of children to the nearest 0.1 kg using a portable electronic weighing machine (TANITA Corporation Japan, Model HD‐305) and height (for children older than 2 years) or length (for children younger than 2 years) to the nearest 0.1 cm using a locally made height and length measuring board. The FSNSP used the 2006 child growth standard of the WHO as a reference population for calculating anthropometric *z*‐scores to evaluate the nutritional status of children (WHO Multicentre Growth Reference Study Group, 2006). Stunting, wasting and underweight of children were defined as the height‐for‐age *z*‐score, weight‐for‐height *z*‐score and weight‐for‐age *z*‐score below −2 SD, respectively, from the median of the WHO reference population.

### 2.3 Exposure variable

We considered HFI as our main exposure variable in this study. The FSNSP followed the HFI Access Scale guideline developed by the Food and Nutrition Technical Assistance project for measuring HFI status (Coates et al., 2007). The FSNSP asked a set of nine questions to mothers to describe the household's experience of food insecurity in the last 30 days from the survey date. These questions captured three main domains of HFI: availability, access and utilization of food. The response to the questions was based on the frequency of occurrence of the situations and was recorded as 0 if the situation never occurred, 1 if the situation occurred rarely (1–2 times), 2 if occurred sometimes (3–10 times) and 3 if occurred often (more than 10 times) during the recall period. Based on the response to the nine defined questions, the FSNSP constructed the HFI score and classified households into four categories: food secure (never occurred), mild food insecure (rarely occurred), moderate food insecure (sometimes occurred) and severe food insecure (often occurred). We combined the last three categories to dichotomize HFI as food secure and food insecure (either mild or moderate or severe) for analysis purposes.

### 2.4 Mediator

**Figure 1:**
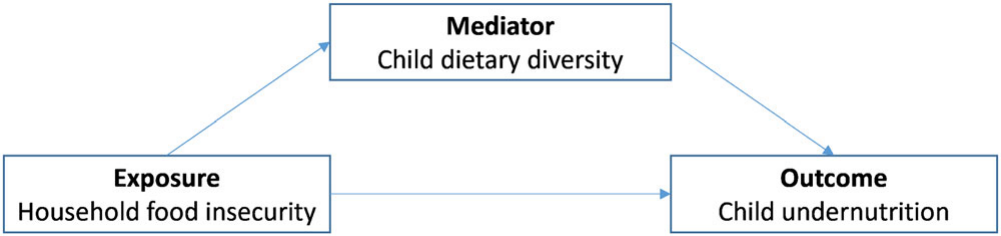
Relationship between household food insecurity, child dietary diversity and child undernutrition

We considered CDD as a mediator in the HFI and child undernutrition pathway (Figure 1). Using the WHO methodology, the FSNSP measured the dietary quality of children 6–23 months of age through the use of a seven‐item scale constructed from the 16 food type categories included in the WHO's standard Infant and Young Child Feeding (IYCF) questionnaires (WHO, 2007, 2010a). The seven‐item scales are grains, roots and tubers; legumes and nuts; dairy products; flesh foods; eggs; vitamin‐A‐rich fruits and vegetables; and other fruits and vegetables. Though standardized IYCF questionnaires do not include older children for dietary assessment, the FSNSP included pre‐school children 24–59 months of age, which allows the FSNSP to assess dietary diversity for children 6–59 months of age. The FSNSP calculated the dietary diversity score of children by adding the score for every seven items ranging from 0 to 7 and categorized the children as consuming an adequately diversified diet (consumed foods from at least four food groups) and inadequately diversified diet (consumed foods from less than four food groups).

### 2.5 Covariates

Based on the literature review and availability of information in the FSNSP data, we considered a set of variables to be adjusted for in the analysis. The variables were selected in such a way that they capture individual, maternal, household and geographical characteristics. These include child's age and sex, childhood morbidity (acute respiratory infection and diarrhea in last 2 weeks), educational status of mother (no education, primary, secondary and higher), maternal age at childbirth, short maternal stature (<145 cm), household size, wealth index of households, place of residence (urban and rural) and administrative division (Barisal, Chittagong, Dhaka, Khulna, Rajshahi, Rangpur and Sylhet). The FSNSP constructed wealth index according to the Demographic and Health Survey method which consists of area‐specific indices that are combined into a national model (Rutstein, 2008). The FSNSP considered household assets and durable goods in constructing the index by applying the principal component analysis technique. The wealth index was then divided into five quintiles: poorest (1st quintile), poorer, middle, richer and richest (5th quintile), each containing an equal proportion of households surveyed.

### 2.6 Statistical analyses

A univariate analysis was conducted to describe the sample characteristics, childhood nutritional status and HFI. Second, a bivariate analysis was applied to understand how the prevalence of child undernutrition varied across HFI. The *χ* ^2^ test was used to check whether the variations in child undernutrition across HFI categories were statistically significant. To investigate the association between HFI and binary child undernutrition outcomes (stunting, wasting and underweight), we performed a multivariable binary logistic regression analysis adjusted for individual, maternal, household and geographical characteristics for each of the outcome variables (e.g., stunting, wasting and underweight) separately. The results of logistic regression analysis were reported in terms of adjusted odds ratio (AOR) along with the 95% confidence interval (CI). All the models were fitted for each year (2012, 2013 and 2014) separately. While analyzing pooled data, time (survey year) was controlled in all the pooled models to adjust the effect of temporal variations in the estimates. Statistical significance was *p* < 0.05 of the association between HFI and child undernutrition in the regression model. Sample weights were adjusted to generalize the estimates for the population of similar characteristics at the national level. Analyses were also adjusted for the strata (geographical region/zone and administrative divisions), primary sampling unit (upazila) and secondary sampling unit (community) to control the variations in the error terms due to the sampling design. All analyses were conducted using the svyset command in Stata to take into account the complex sampling design. The details of the svyset command are explained in the Stata manual (StataCorp, 2016). The absence of multicollinearity was confirmed through the lower value of the variance inflation factor (<3) while fitting multivariable regression models.

To understand the extent of how CDD mediates in the HFI and child undernutrition pathways, causal mediation analysis was utilized for binary outcomes, exposure and mediator. The mediator was selected so that it is influenced by the exposure and influenced the outcome. The Stata built‐in ‘*paramed*’ command, developed for a binary outcome, exposure and mediator, was used to conduct the mediation analysis and calculate the percentage of the total effect that the CDD mediates in the HFI and child undernutrition relationship. This command provides the natural direct and indirect effects of exposure on outcomes in terms of odds ratio to understand the effect sizes. More details about the command can be found elsewhere (Emsley & Liu, 2013). All the analyses were repeated for three seasons, both for pooled and year‐specific investigation, separately to understand the extent of how the estimates varied across seasons. Data were analyzed in Stata (version 16).

### 2.7 Ethical clearance

The FSNSP obtained ethical clearance from the European Union. Verbal informed consent was taken from FSNSP study participants. In the case of children, consent was taken from the mother or primary caregiver. The outcome of the consent procedure was recorded in the consent form by the interviewer. The FSNSP data is anonymous and can be accessed by external users upon request.

## 3 RESULTS

### 3.1 Sample characteristics

Data were analyzed for 26,353 youngest children 6–59 months of age (mean age: 30.4 months). Nearly half were female (48.0%). One in 10 children (10.7%) suffered from diarrhea in the last 2 weeks before the survey. Among mothers of the indexed child, nearly one in five (18.1%) had no education, and more than one in 10 were short (12.3%). On average, mothers were 24 years of age while delivering the indexed child. On average, the households where the child lived had five (4.96) members. More than a quarter (27.5%) of the children belonged to the poorest households. These results were nearly similar across seasons (Table 1), across years and seasons within years (Table S1).

**Table 1:** Child, maternal and household characteristics during 2012–2014

	Overall	Across season		
		Post‐Aman	Monsoon	Post‐Aus
Dependent variable				
Child stunting (%)	36.0	35.1	35.5	37.9
Child underweight (%)	31.9	28.8	34.5	32.1
Child wasting (%)	11.6	9.5	14.4	10.4
Exposure variable (%)				
Household food insecurity	37.9	39.5	36.4	38.0
Mediator variable (%)				
Inadequate DD (child consumed <4 of 7 food groups)	46.2	49.8	39.2	51.2
*Covariates*				
Children's individual characteristics				
Age (months)	30.4 ± 0.17	30.2 ± 0.32	30.3 ± 0.28	30.9 ± 0.23
Sex, % female	48.0	47.5	48.8	47.7
Acute respiratory infection (%)	1.8	1.5	1.4	2.5
Diarrhea (%)	10.7	11.2	11.8	8.7
Mother's characteristics				
Age at childbirth (years)	24.04 ± 0.05	24.06 ± 0.10	24.05 ± 0.08	24.0 ± 0.09
Education (%)				
No education	18.1	18.0	17.8	18.5
Primary	32.0	31.1	31.9	33.1
Secondary	44.0	45.0	43.7	43.4
Higher	5.9	5.9	6.6	5.0
Short stature (<145 cm) (%)	12.3	11.4	12.4	13.1
Household characteristics				
Household size, *n*	4.96 ± 0.02	4.94 ± 0.04	5.02 ± 0.04	4.91 ± 0.03
Wealth quintiles (%)				
Poorest	27.5	26.1	26.7	30.0
Poorer	21.0	19.7	20.6	22.9
Middle	18.3	17.5	20.1%	16.9
Richer	17.2	19.4	16.7	15.3
Richest	16.0	17.3	15.8	14.8
Geographical locations				
Rural (%)	87.2	87.2	87.0	87.4
Division (%)				
Rajshahi	11.6	11.0	12.2	11.4
Khulna	10.8	8.5	10.9	13.3
Barisal	6.4	6.8	6.6	5.6
Dhaka	32.9	34.8	33.8	29.4
Sylhet	6.5	5.6	7.4	6.1
Chittagong	17.7	19.5	15.7	18.4
Rangpur	14.2	13.8	13.2%	15.8

Abbreviation: DD, dietary diversity.

### 3.2 Nutritional status of children

Overall, 36.0%, 11.6% and 31.9% of children were stunted, wasted and underweight respectively. The prevalence of wasting and underweight varied significantly across seasons, with the highest prevalence of underweight (34.5%) and wasting (14.4%) recorded during the Monsoon season. Although the prevalence of stunting was highest during the Post‐Aus season (37.9%) compared with other seasons. However, seasonal variation in stunting prevalence was not statistically significant (*χ* ^2^: 16.1179, *p* = 0.0739) (Table 1). No significant variations in the prevalence of child undernutrition were observed across survey years, but variations were seen across seasons within survey years, with childhood wasting and underweight were highest during the Monsoon season in 2013 and 2014 (Figure S2).

### 3.3 HFI

Overall, nearly two of five children (37.9%) lived in food‐insecure households. The prevalence of HFI remained similar across seasons from 36.4% in the Monsoon season to 39.5% Post‐Aman season. These variations were not statistically significant. Over time, the prevalence of HFI significantly declined from 57.0% in 2012 to 24.6% in 2014 (Figure S2).

### 3.4 CDD

**Figure 2:**
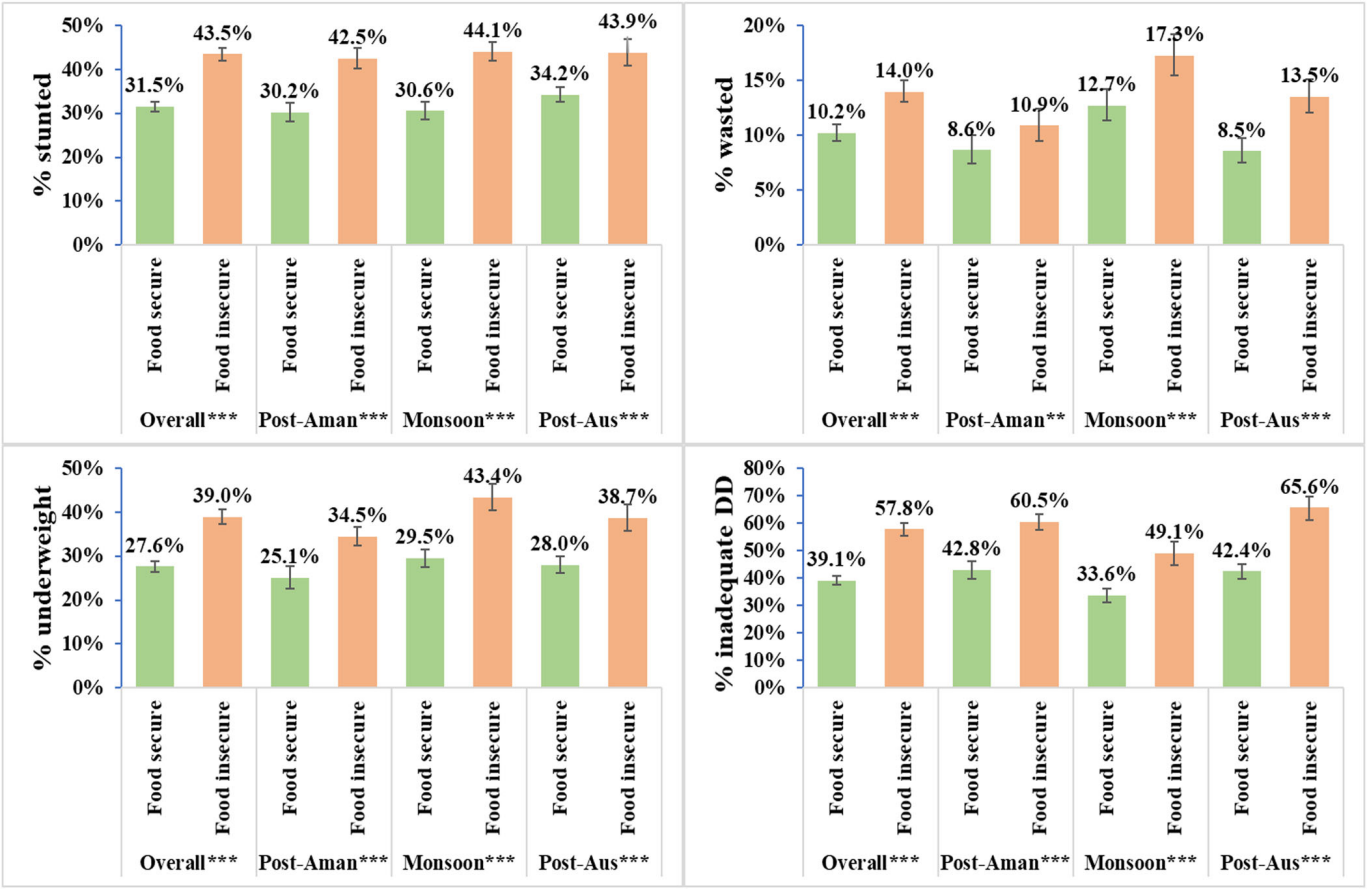
Prevalence of undernutrition and consumption of minimum diversified diet among children 6–59 months of age by household food insecurity in Bangladesh during 2012–2014. ***p < 0.001, **p < 0.01 and *p < 0.05.

The average CDD score (3.65) was below the cut‐off of the minimum acceptable level (four out of seven) for an adequate diversified diet. Nearly half of the children (46.2%) consumed an inadequate diversified diet. The prevalence of inadequate CDD varied significantly across seasons, with the highest prevalence observed during the Post‐Aus season (51.2%) (Table 1). The prevalence of inadequate CDD significantly (*p* < 0.05) decreased over the survey years. Further, the prevalence of inadequate CDD also varied across seasons when tested within each year, with the prevalence of inadequate CDD remaining topping during the Post‐Aus season in 2012 and 2013 but during the Post‐Aman season in 2014 (Figure S2). The prevalence of inadequate CDD was greater in food‐insecure households than their counterparts in food‐secure households, irrespective of survey years and seasons (Figure 2).

### 3.5 HFI and child undernutrition

The prevalence of child undernutrition varied significantly across HFI (Figure 2), with the prevalence of child undernutrition was greater in food‐insecure than in food‐secure households (stunting: 43.5% vs. 31.5%; wasting: 14.0% vs. 10.2%; underweight: 39.0% vs. 27.6%). A significantly greater prevalence of child undernutrition in food‐insecure households was also observed across seasons (Figure 2), across survey years and across seasons within the survey years (Figure S3). However, there were some exceptions, with no statistically significant association between childhood wasting and HFI was detected during the Post‐Aman season in 2013 and 2014 (Figure S3).

**Figure 3:**
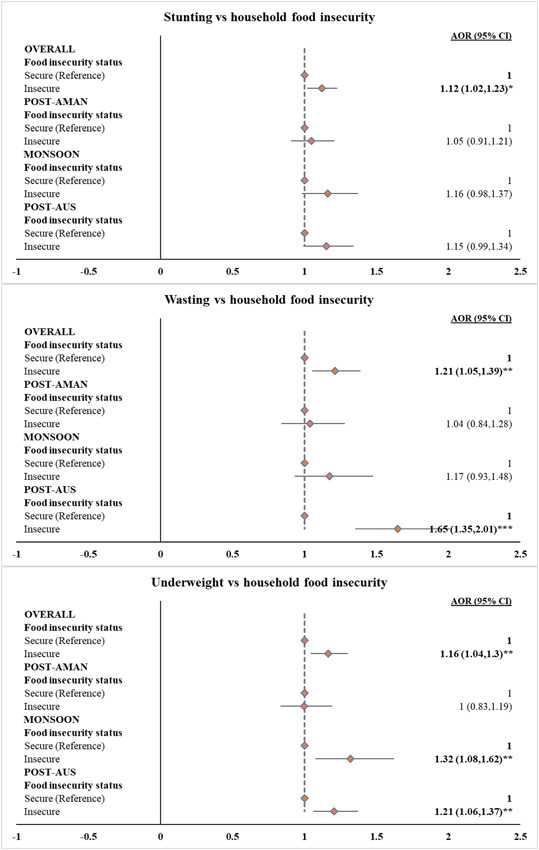
Association between household food insecurity and child undernutrition in Bangladesh during 2012–2014. ***p < 0.001, **p < 0.01 and *p < 0.05. All the models were separately fitted at an overall level and for all three seasons. All the models were adjusted for the age of child in months, child sex, child morbidity (acute respiratory infections and diarrhea), mother's education, maternal age at birth, maternal stature, household size, wealth quintiles of households, place of residence (rural and urban) and division. AOR, adjusted odds ratio; CI, confidence interval.

The results of logistic regression analyses adjusted for potential covariates presented in Figure 3 depict that compared with children of food‐secure households, children of food‐insecure households were 12%, 21% and 16% more likely to be stunted (AOR: 1.12; 95% CI: 1.02–1.23; *p* < 0.05), wasted (AOR: 1.21; 95% CI: 1.05–1.39; *p* < 0.01) and underweight (AOR: 1.16; 95% CI: 1.04–1.30; *p* < 0.01), respectively. These associations varied across seasons. During the Post‐Aus season, the odds of wasting (AOR: 1.65; 95% CI: 1.35–2.01; *p* < 0.001) and underweight (AOR: 1.21; 95% CI: 1.06–1.37; *p* < 0.01) were greater among children of food‐insecure than food‐secure households. Also, HFI was associated with greater odds of childhood underweight (AOR: 1.32; 95% CI: 1.08–1.62; *p* < 0.01) during the Monsoon season. HFI was not significantly associated with all forms of child undernutrition during the Post‐Aman season and with stunting across all seasons (Figure 3). The association between HFI and child undernutrition further varied across survey years and seasons within each survey year when investigated separately (Table S2).

### 3.6 Mediation effect of CDD in the association between HFI and child undernutrition

The results of the mediation analysis are presented in Table 2. Overall, CDD showed a small but significant indirect effect on child underweight (AOR: 1.01; 95% bias‐corrected CI: 1.002–1.02) and mediated 6.1% of the total effect of HFI on underweight. The indirect effect of CDD on underweight was largest during the Post‐Aman season when 23.2% of the total effect of HFI on underweight was mediated by CDD. These mediation effects also varied across survey years and seasons within years. Repeating the analysis across survey years showed a slight but significant indirect effect of CDD on underweight in 2012 (AOR: 1.01; 95% bias‐corrected CI: 1.0003–1.02) and 2013 (AOR: 1.01; 95% bias‐corrected CI: 1.004–1.04), when CDD mediated 5.4% and 9.9% of the total effect of HFI on underweight, respectively. In addition, CDD mediated 39.7% of the total effect of HFI on wasting during Post‐Aman season in 2014 and 13.7% of the total effect on underweight during the same season in 2012. No significant indirect effect of CDD in the association between HFI and child stunting was observed across years and seasons within years (Table S3).

**Table 2:** Mediation effect of inadequate dietary diversity in the association between household food insecurity and child undernutrition in Bangladesh during 2012–2014

	Outcome: Stunting			
	Total effect	Natural direct effect	Natural indirect effect	Absolute mediation effect (%)
	AOR (95% CI)	AOR (95% CI)	AOR (95% CI)	
Overall	**1.12 (1.05–1.19)**	**1.12 (1.05–1.19)**	1.00 (0.99–1.01)	0.2
Post‐Aman	1.10 (0.99–1.23)	1.09 (0.98–1.22)	1.01 (0.997–1.02)	10.1
Monsoon	**1.13 (1.0–1.24)**	**1.13 (1.02–1.25)**	0.99 (0.98–1.0)	−6.1
Post‐Aus	**1.15 (1.03–1.33)**	**1.15 (1.03–1.33)**	1.00 (0.98–1.01)	−2.4

	**Outcome: Wasting**			
	**Total effect**	**Natural direct effect**	**Natural indirect effect**	**Absolute mediation effect (%)**
	**AOR (95% CI)**	**AOR (95% CI)**	**AOR (95% CI)**	
Overall	**1.18 (1.08–1.31)**	**1.18 (1.07–1.30)**	1.00 (0.99–1.01)	1.4
Post‐Aman	1.09 (0.92–1.30)	1.08 (0.92–1.30)	1.01 (0.996–1.03)	15.1
Monsoon	1.11 (0.97–1.28)	1.11 (0.97–1.28)	1.01 (0.996–1.02)	7.0
Post‐Aus	**1.53 (1.30–1.87)**	**1.53 (1.30–1.86)**	1.00 (0.98–1.02)	0.4

	**Outcome: Underweight**			
	**Total effect**	**Natural direct effect**	**Natural indirect effect**	**Absolute mediation effect (%)**
	**AOR (95% CI)**	**AOR (95% CI)**	**AOR (95% CI)**	
Overall	**1.15 (1.08–1.23)**	**1.14 (1.07–1.22)**	**1.01 (1.002–1.02)**	6.1
Post‐Aman	1.08 (0.97–1.21)	1.06 (0.95–1.19)	**1.02 (1.01–1.03)**	23.2
Monsoon	**1.22 (1.10–1.36)**	**1.21 (1.10–1.34)**	1.01 (0.998–1.02)	4.1
Post‐Aus	**1.21 (1.06–1.35)**	**1.20 (1.06–1.33)**	1.01 (0.997–1.02)	5.1

*Note*: Bold values denote statistical significance (no “0” values in the CI).

Abbreviations: AOR, adjusted odds ratio; CI, bias‐corrected confidence interval.

## 4 DISCUSSION

This study demonstrates two major findings to enrich the existing literature. First, HFI is significantly associated with greater odds of child undernutrition. However, the association is mixed across survey years with the association of HFI and stunting significant in 2013, with wasting in 2012 and 2014 and with underweight in 2012 and 2013, and across seasons within years with a significant association of HFI observed in Post‐Aus harvest season with stunting in 2014 and with wasting in 2012 and 2014, while HFI was associated with underweight during the Monsoon and Post‐Aus harvest seasons in 2012. Second, CDD has a considerable mediating effect in the association between HFI and child underweight but not with other forms. This mediating effect further varied across seasons, survey years and seasons within survey years. The mediation effect of CDD in HFI and underweight association was noticed in Post‐Aman harvest season when examined from pooled data. However, analysis across survey years and seasons within survey years further altered the mediation results. These findings suggest that seasonality should be considered with caution while designing and implementing agricultural and/or food‐based interventions to combat child undernutrition in Bangladesh.

Our findings on the positive association between HFI and child undernutrition coincide with previous research studies (Hackett et al., 2009; Matheson et al., 2002; Saha et al., 2009). However, no significant association with child undernutrition has also been found (Alaimo et al., 2001; Bhattacharya et al., 2004; Kaiser et al., 2002; Osei et al., 2010). These mixed associations could be due to several reasons including but not limited to the changes in the study contexts, sample sizes and agricultural seasons among these studies. HFI can directly or indirectly affect child undernutrition in multiple pathways. Saha et al. (2009) showed a fall in the growth of children living in food‐insecure households. HFI may trigger maternal anxiety and depressive symptoms (Hadley & Patil, 2006; Whitaker et al., 2006) that result directly in growth faltering of children (Harpham et al., 2005) or indirectly through unpleasant parenting with a lack of care for children (McLearn et al., 2006).

Seasonal variation in HFI, dietary diversity and undernutrition of children coincides with previous research (Belayneh et al., 2021), likely due to the seasonal variation in agricultural production. As reported in previous studies (Agbadi et al., 2017; N. B. Ali et al., 2019), we found lower CDD among children of food‐insecure households during Monsoon season, with higher rates of inadequate CDD among food‐insecure households during Post‐Aman and Post‐Aus seasons. Our findings demonstrate that the effect of HFI on child undernutrition persisted during the Post‐Aus harvest season, typically considered the lean period when households become prone to starvation for food (Raihan et al., 2018). As a result, childhood consumption of foods from diversified food groups is largely affected during this season (Madan et al., 2018). Consumption of inadequately diversified foods is likely to contribute to a lower intake of micronutrients among children (Institute of Public Health Nutrition Ministry of Health and Family Welfare Government of the People's Republic of Bangladesh, 2015). Lack of micronutrient‐rich food impedes the growth of infants and children, which further leads to reduced protective power from contaminating diseases and increases the risk of being hospitalized (Cook et al., 2004). This may further result in considerable weight loss in children.

Not surprisingly, the association between HFI and childhood stunting that was drawn from both pooled and year‐specific data remained statistically insignificant across seasons. Childhood stunting depicts the chronic nutritional status of children that may result from the longer effect of harmful factors including HFI. However, our study noted no remarkable variation in HFI across seasons, even when investigated across individual survey periods.

The inconsistent mediation effect of CDD that varied approximately from null to a one‐quarter percentage in HFI and child undernutrition pathways was also not surprising due to the seasonal fluctuations in the prevalence of these indicators and their interrelationships. Our findings on the mediation effect of CDD in the association between HFI and child undernutrition were supported by a previous study conducted by Chandrashekhar et al. (2017) in India, but contradict the findings of other studies conducted by Disha and colleagues in Bangladesh, Ethiopia and Vietnam (D. Ali et al., 2013). The deviations in the findings may be due to the lack of consideration of seasonality in previous research. However, our season‐specific analysis showed that the mediating role of CDD varied across seasons, with the largest contribution of CDD detected during the Post‐Aman season when children suffered from having adequately diversified foods. In Bangladesh, *Boro* rice is the main paddy crop harvested during the Monsoon season. After this season, *Aus* rice is cultivated in some parts of Bangladesh before starting the *Aman* season. As a result, there is a gap between the Monsoon and Post‐Aman harvest season, likely causing a greater chance of inadequate CDD practices during Post‐Aman season. Our findings demonstrate that the association between HFI and child undernutrition and the role of CDD in this relationship is largely modifiable subject to seasonality and hence provides the basis for further thought while designing agriculture/food‐based nutrition interventions in Bangladesh. Seasonal variation in the diet, energy and nutrient intake of children has also been documented (Huong et al., 2014). However, seasonality should be considered with caution while designing nutrition interventions given the insignificant association of HFI, CDD and child undernutrition across seasons when investigated annually.

To our knowledge, this is the first‐ever study testing the season‐specific role of CDD in this pathway in Bangladesh. FSNSP data is the only source of nationally representative data on both food and nutrition across three seasons in Bangladesh. However, the cross‐sectional nature of FSNSP data limits the inference of causal association. Another limitation of this study is to infer stunting, which may be the result of a chronic food crisis or break of linear growth while the measurement of HFI was based on household's experience of food insufficiency/shortage in the 30 days before the survey. Although FSNSP evaluated HFI and anthropometry three times within the same year through repeated cross‐sectional surveys, longitudinal studies with longer duration and frequent follow‐up of the same households/participants may better explain the relationship of HFI with chronic nutritional status of children, which was out of scope for our case. We were unable to adjust the effect of natural hazards (e.g., floods, cyclones, storm, etc.) due to a lack of information in the FSNSP data. The FSNSP team spent 4–6 weeks during each of the seasons in the field to collect the data, which may not have captured the full coverage of the seasons while collecting data. However, FSNSP collected data roughly similar time period in each year, maintaining consistent timing of data collection across years and the ability to compare estimates across seasons. Lastly, our analysis included the last round of the FSNSP data, which was collected in 2014. This was the most recent nationally representative surveillance data. However, a shorter version of the FSNSP was conducted by the James P. Grant School of Public Health in 2019. Therefore, we recommend further investigation to ascertain the seasonal effect in the HFI and child undernutrition association using this data if available publicly.

## 5 CONCLUSION

Our study concludes that HFI is seasonally associated with child undernutrition and is mediated by CDD in Bangladesh. These findings recommend consideration of seasonality while designing appropriate food‐based interventions to downgrade child undernutrition in Bangladesh. Appropriate interventions to enhance the nutritional status of children should be implemented to ensure the sustainability of food security, especially in the lean season. The promotion of food‐based approaches through homestead food production incorporating climate‐smart agriculture technology could be useful to alleviate food insecurity and reduces child undernutrition (Iannotti et al., 2010). As CDD has been explored to have mediation effects in the pathways between HFI and child undernutrition, emphasis needs to be given to home‐based year‐round diversified food production to ensure childhood consumption of diversified diets for their uninterrupted growth.

## AUTHOR CONTRIBUTIONS

*Conceptualization*: Md. Mehedi Hasan. *Methodology*: Md. Mehedi Hasan and Abdul Kader. *Software*: Md. Mehedi Hasan. *Formal analysis*: Md. Mehedi Hasan. *Data curation*: Md. Mehedi Hasan. *Writing – original draft preparation*: Md. Mehedi Hasan. *Writing – review and editing*: Abdul Kader, Chowdhury Abdullah Al Asif and Aminuzzaman Talukder. *Visualization*: Md. Mehedi Hasan. All authors have read and approved this version for journal submission.

## CONFLICT OF INTEREST

The authors declare no conflict of interest.

## Supporting information

Supporting information.

## Data Availability

The data that support the findings of this study are available from the corresponding author upon reasonable request.
