# Supplementary material for: Seasonal variation in the association between household food insecurity and child undernutrition in Bangladesh: Mediating role of child dietary diversity
Source: Matern Child Nutr. 2022 Dec 7;19(2):e13465. doi: 10.1111/mcn.13465 (PMC10019058; doi:10.1111/mcn.13465)
Supplement: Supplementary file 1 — Supporting information. [file MCN-19-e13465-s001.docx]

**Supplementary**

**Figure S1:** Selection of sample, Food Security Nutritional Surveillance Project 2012-2014

**
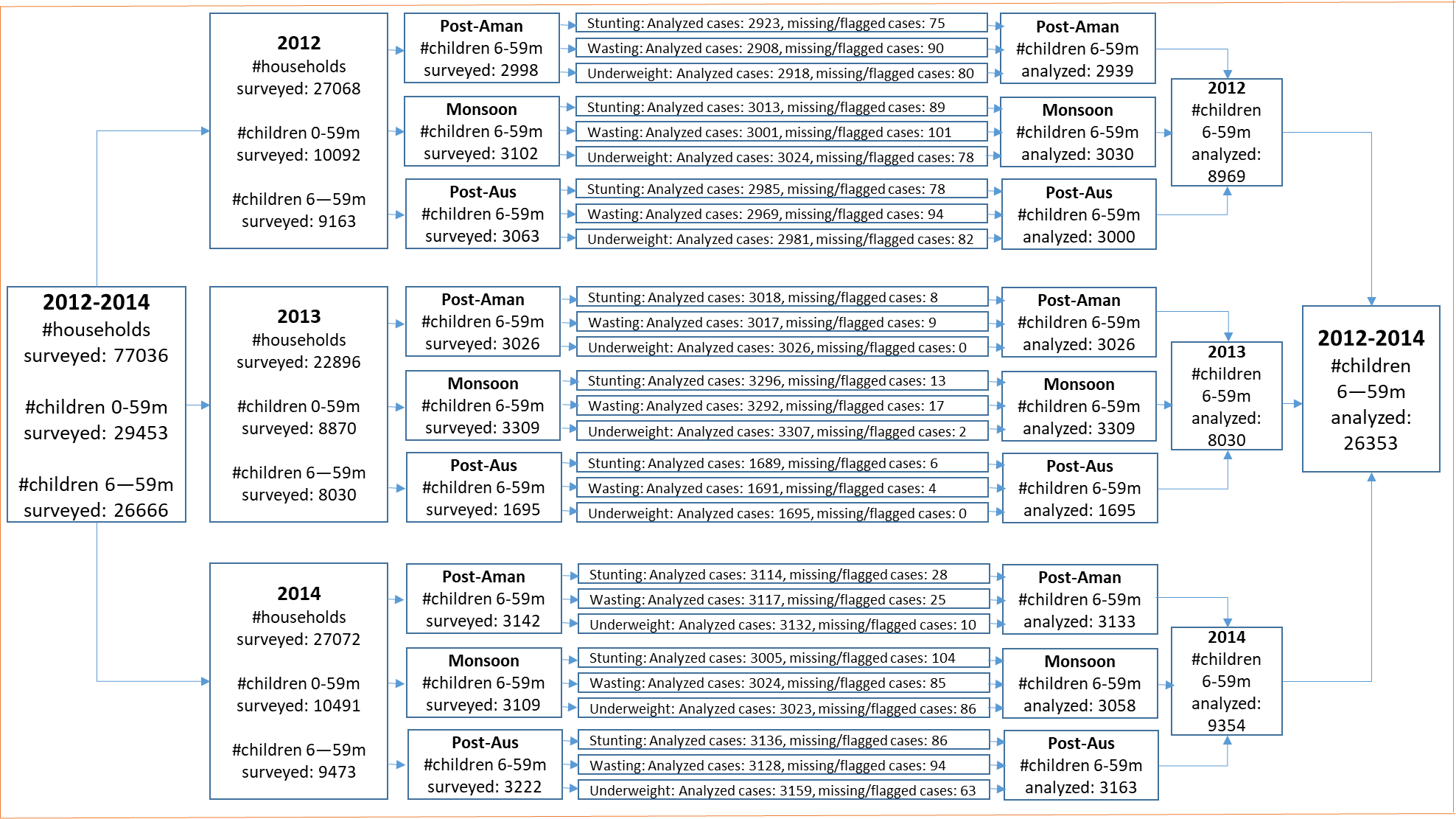
**

**Table S1:** Child, maternal and household characteristics for each year during 2012-2014

|  | **2012** | | | | **2013** | | | | **2014** | | | |
| --- | --- | --- | --- | --- | --- | --- | --- | --- | --- | --- | --- | --- |
|  | **Overall** | **Post-Aman** | **Monsoon** | **Post-Aus** | **Overall** | **Post-Aman** | **Monsoon** | **Post-Aus** | **Overall** | **Post-Aman** | **Monsoon** | **Post-Aus** |
| ***Dependent variable*** |  |  |  |  |  |  |  |  |  |  |  |  |
| Child stunting, % | 37.3% | 36.9% | 36.6% | 38.4% | 35.2% | 33.0% | 36.9% | 35.9% | 35.5% | 35.4% | 33.2% | 38.3% |
| Child underweight, % | 33.8% | 32.8% | 34.9% | 33.7% | 30.7% | 27.5% | 34.5% | 28.5% | 31.2% | 25.8% | 34.2% | 32.1% |
| Child wasting, % | 11.7% | 10.6% | 13.7% | 11.0% | 11.5% | 9.3% | 14.4% | 9.3% | 11.7% | 8.5% | 14.9% | 10.4% |
| ***Exposure variable, %*** |  |  |  |  |  |  |  |  |  |  |  |  |
| Household food insecurity | 57.0% | 61.9% | 55.5% | 53.7% | 32.0% | 29.5% | 33.4% | 33.9% | 24.6% | 25.8% | 24.1% | 24.3% |
| ***Mediator variable, %*** |  |  |  |  |  |  |  |  |  |  |  |  |
| Inadequate DD (Child consumed <4  of 7 food groups) | 48.1% | 50.6% | 37.4% | 55.8% | 47.5% | 49.6% | 43.9% | 51.8% | 43.2% | 49.1% | 36.3% | 46.4% |
| ***Covariates*** |  |  |  |  |  |  |  |  |  |  |  |  |
| **Children's characteristics** |  |  |  |  |  |  |  |  |  |  |  |  |
| Age, m | 30.9±0.37 | 30.6±0.84 | 30.6±0.65 | 31.5±0.36 | 30.0±0.24 | 30.1±0.38 | 29.7±0.39 | 30.5±0.48 | 30.4±0.22 | 29.9±0.32 | 30.5±0.39 | 30.6±0.38 |
| Sex, % female | 46.9% | 46.0% | 47.5% | 47.1% | 48.8% | 48.9% | 48.5% | 49.0% | 48.5% | 47.5% | 50.0% | 47.7% |
| ARI, % | 2.3% | 2.0% | 1.8% | 3.1% | 1.3% | 1.2% | 0.9% | 2.6% | 1.7% | 1.4% | 1.6% | 1.9% |
| Diarrhea, % | 12.7% | 13.0% | 15.9% | 9.4% | 10.1% | 11.2% | 10.1% | 8.0% | 9.3% | 9.3% | 10.2% | 8.3% |
| **Mother's characteristics** |  |  |  |  |  |  |  |  |  |  |  |  |
| Age at child birth, y | 24.07±0.11 | 24.12±0.22 | 24.08±0.20 | 24.0±0.13 | 24.0±0.09 | 23.98±0.19 | 23.99±0.10 | 24.05±0.21 | 24.04±0.04 | 24.08±0.15 | 24.09±0.13 | 23.95±0.14 |
| Education, % |  |  |  |  |  |  |  |  |  |  |  |  |
| No education | 18.8% | 17.8% | 18.6% | 20.0% | 17.1% | 16.9% | 17.9% | 15.8% | 18.2% | 19.9% | 16.9% | 18.3% |
| Primary | 31.0% | 30.3% | 31.9% | 30.9% | 29.7% | 29.5% | 29.5% | 30.6% | 35.3% | 34.2% | 34.7% | 37.1% |
| Secondary | 44.8% | 47.0% | 42.4% | 45.1% | 47.3% | 46.8% | 47.7% | 47.7% | 39.9% | 40.1% | 40.5% | 39.0% |
| Higher | 5.4% | 4.9% | 7.1% | 4.0% | 5.8% | 6.8% | 5.0% | 5.9% | 6.6% | 5.8% | 7.9% | 5.6% |
| Short stature (<145 cm), % | 12.5% | 10.1% | 13.4% | 13.9% | 12.0% | 11.9% | 12.8% | 10.5% | 12.4% | 12.3% | 11.5% | 13.5% |
| **Household characteristics** |  |  |  |  |  |  |  |  |  |  |  |  |
| Household size, n | 4.9±0.03 | 4.9±0.06 | 4.94±0.07 | 4.87±0.05 | 5.0±0.04 | 4.95±0.08 | 5.06±0.06 | 4.94±0.08 | 4.99±0.05 | 4.96±0.07 | 5.04±0.10 | 4.94±0.05 |
| Wealth quintiles, % |  |  |  |  |  |  |  |  |  |  |  |  |
| Poorest | 28.0% | 27.3% | 26.3% | 30.3% | 27.5% | 25.9% | 27.4% | 31.0% | 26.9% | 25.0% | 26.4% | 29.1% |
| Poorer | 21.8% | 21.0% | 21.6% | 22.9% | 20.3% | 19.2% | 21.1% | 20.7% | 20.8% | 18.8% | 19.5% | 24.1% |
| Middle | 17.9% | 17.3% | 18.7% | 17.6% | 18.4% | 17.2% | 20.6% | 15.8% | 18.7% | 18.1% | 20.7% | 16.8% |
| Richer | 16.5% | 17.0% | 16.7% | 15.9% | 18.6% | 21.2% | 17.6% | 15.9% | 16.6% | 20.0% | 15.9% | 14.5% |
| Richest | 15.8% | 17.5% | 16.7% | 13.3% | 15.1% | 16.6% | 13.3% | 16.6% | 17.1% | 18.1% | 17.6% | 15.5% |
| **Geographical locations** |  |  |  |  |  |  |  |  |  |  |  |  |
| Rural, % | 86.0% | 85.4% | 83.1% | 89.3% | 90.8% | 90.1% | 91.8% | 89.9% | 85.2% | 86.0% | 85.5% | 84.1% |
| Division, % |  |  |  |  |  |  |  |  |  |  |  |  |
| Rajshahi | 11.9% | 10.5% | 12.7% | 12.6% | 10.3% | 8.4% | 10.6% | 13.2% | 12.4% | 14.6% | 13.5% | 9.3% |
| Khulna | 10.4% | 9.0% | 10.5% | 11.7% | 9.4% | 9.3% | 8.5% | 12.0% | 12.4% | 7.0% | 13.6% | 15.6% |
| Barisal | 5.2% | 5.7% | 5.3% | 4.7% | 4.9% | 5.7% | 6.4% | 0.0% | 8.7% | 9.2% | 7.8% | 9.2% |
| Dhaka | 35.9% | 35.6% | 35.7% | 36.3% | 35.5% | 37.1% | 37.0% | 28.6% | 27.8% | 31.3% | 29.4% | 23.0% |
| Sylhet | 5.9% | 5.7% | 5.8% | 6.1% | 4.7% | 4.8% | 4.9% | 4.3% | 8.5% | 6.6% | 11.1% | 6.9% |
| Chittagong | 19.1% | 21.5% | 20.1% | 15.6% | 19.9% | 18.4% | 19.6% | 23.3% | 14.5% | 18.3% | 8.5% | 18.6% |
| Rangpur | 11.6% | 12.0% | 9.8% | 13.0% | 15.3% | 16.4% | 13.0% | 18.5% | 15.6% | 13.0% | 16.1% | 17.3% |

**Figure S2:** Changes in the prevalence of household food insecurity, child dietary diversity and nutritional status by year and season


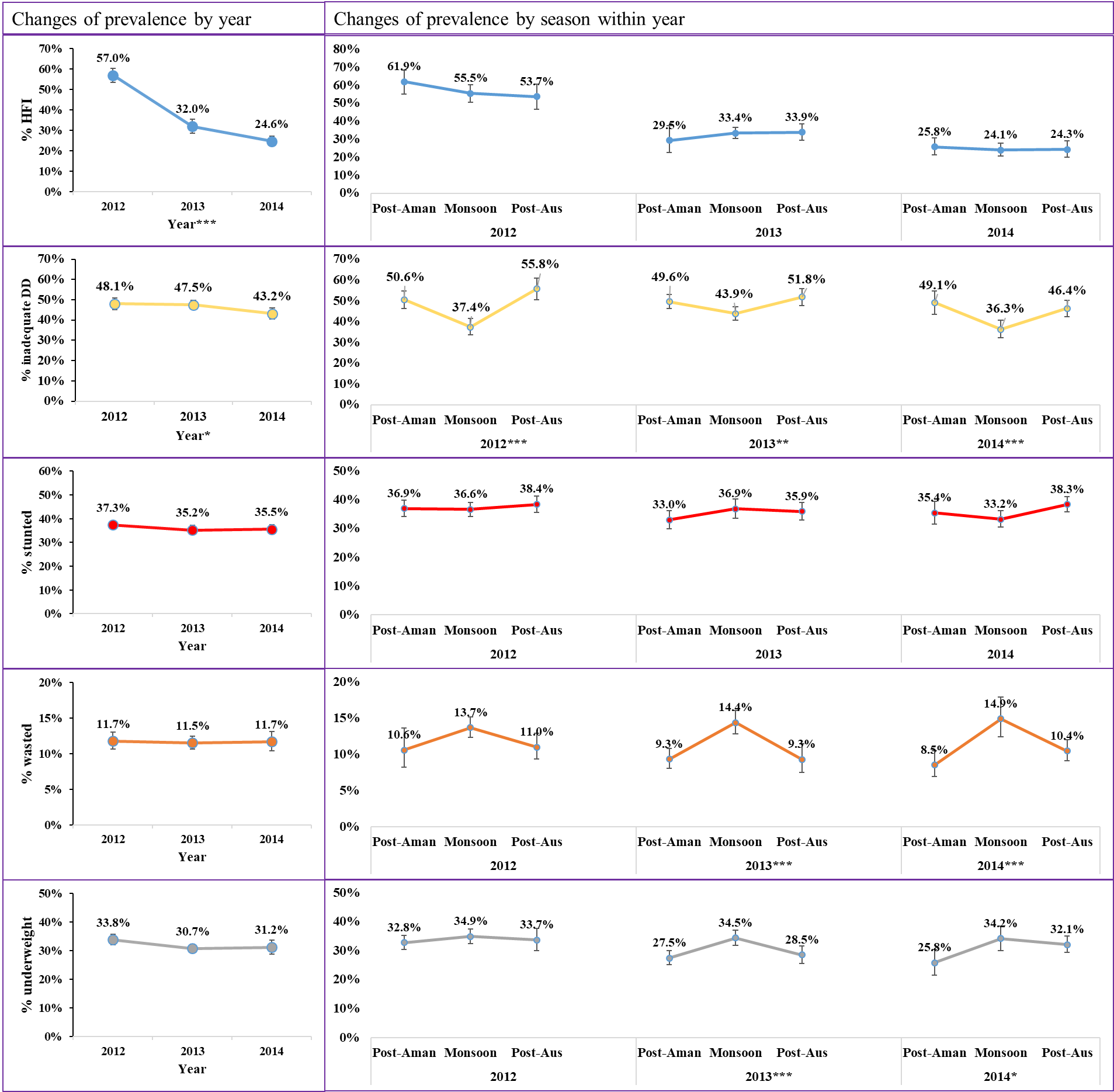


**Note:** HFI= Household food insecurity, DD= dietary diversity, ***, ** and * denotes statistically significant when p<0.001, p<0.01 and p<0.05 respectively.

**Figure S3:** Prevalence of undernutrition and consumption of minimum diversified diet among children 6-59 months of age by household food insecurity in Bangladesh across survey years


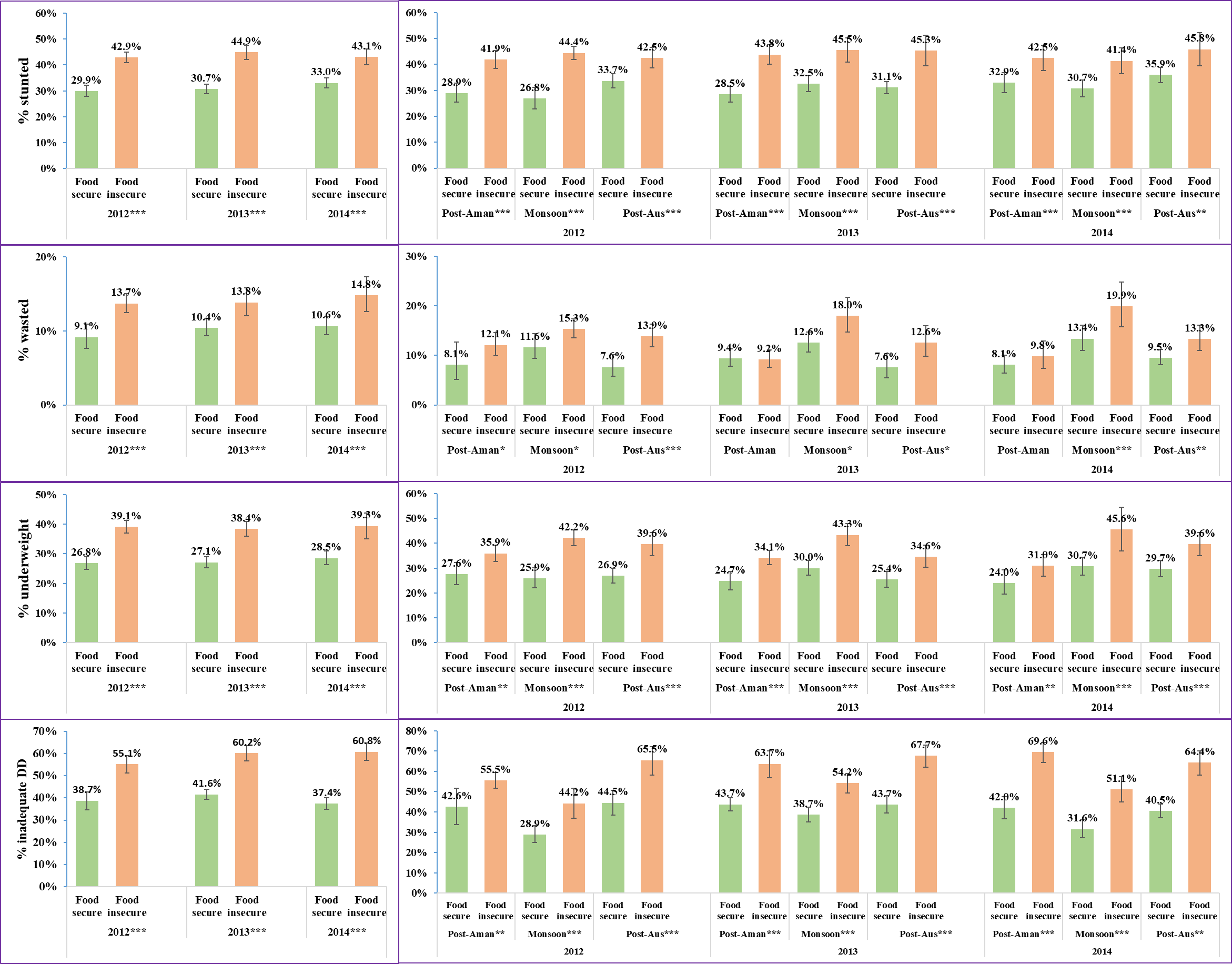


**Note:** DD= dietary diversity, ***, ** and * denotes statistically significant when p<0.001, p<0.01 and p<0.05 respectively.

**Table S2:** Association between household food insecurity and child undernutrition in Bangladesh across survey years

|  | **Adjusted odds ratio with 95% confidence intervals** | | | | | | | | | | |
| --- | --- | --- | --- | --- | --- | --- | --- | --- | --- | --- | --- |
|  | **Stunting** | | |  | **Wasting** | | |  | **Underweight** | | |
|  | **2012** | **2013** | **2014** |  | **2012** | **2013** | **2014** |  | **2012** | **2013** | **2014** |
| ***Overall*** |  |  |  |  |  |  |  |  |  |  |  |
| **Food insecurity status** |  |  |  |  |  |  |  |  |  |  |  |
| Food secure (RC) |  |  |  |  |  |  |  |  |  |  |  |
| Food insecure | 1.11 (0.96-1.29) | **1.18 (1.03-1.37)** | 1.06 (0.88-1.29) |  | **1.27 (1.04-1.55)** | 1.13 (0.86-1.5) | **1.27 (1-1.61)** |  | **1.18 (1.02-1.36)** | **1.2 (1.01-1.42)** | 1.16 (0.9-1.49) |
| ***Post-Aman*** |  |  |  |  |  |  |  |  |  |  |  |
| **Food insecurity status** |  |  |  |  |  |  |  |  |  |  |  |
| Food secure (RC) |  |  |  |  |  |  |  |  |  |  |  |
| Food insecure | 1.05 (0.84-1.31) | 1.2 (0.93-1.56) | 0.9 (0.67-1.21) |  | 1.25 (0.91-1.71) | 0.88 (0.59-1.31) | 0.88 (0.63-1.23) |  | 0.99 (0.7-1.4) | 1.16 (0.94-1.43) | 0.87 (0.67-1.14) |
| ***Monsoon*** |  |  |  |  |  |  |  |  |  |  |  |
| **Food insecurity status** |  |  |  |  |  |  |  |  |  |  |  |
| Food secure (RC) |  |  |  |  |  |  |  |  |  |  |  |
| Food insecure | 1.37 (1-1.88) | 1.19 (0.96-1.48) | 0.95 (0.65-1.38) |  | 1.01 (0.71-1.44) | 1.19 (0.78-1.8) | 1.39 (0.98-1.98) |  | **1.4 (1.13-1.74)** | 1.31 (0.98-1.76) | 1.39 (0.84-2.29) |
| ***Post-Aus*** |  |  |  |  |  |  |  |  |  |  |  |
| **Food insecurity status** |  |  |  |  |  |  |  |  |  |  |  |
| Food secure (RC) |  |  |  |  |  |  |  |  |  |  |  |
| Food insecure | 1.01 (0.81-1.25) | 1.27 (0.98-1.65) | **1.33 (1.02-1.75)** |  | **1.88 (1.33-2.66)** | 1.57 (0.93-2.63) | **1.51 (1.1-2.07)** |  | **1.26 (1.09-1.46)** | 1.07 (0.79-1.45) | 1.23 (0.95-1.6) |

**Note:** RC= Reference category. Bold values denote statistically significant at 5% level (p-value<0.05)

**Table S3:** Mediation effect of inadequate dietary diversity in the association between household food insecurity and child undernutrition in Bangladesh during 2012-2014

|  | **Outcome: Stunting** | | | |
| --- | --- | --- | --- | --- |
|  | **Total effect** | **Natural Direct Effect** | **Natural Indirect Effect** | **Absolute mediation effect, %** |
|  | **AOR (95% CI^1^)** | **AOR (95% CI^1^)** | **AOR (95% CI^1^)** |  |
| **2012** |  |  |  |  |
| Overall | 1.09 (0.99-1.22) | 1.09 (0.97-1.21) | 1.00 (0.99-1.01) | 3.6% |
| Post-Aman | 1.08 (0.88-1.32) | 1.07 (0.87-1.31) | 1.01 (0.999-1.03) | 12.7% |
| Monsoon | 1.15 (0.97-1.37) | 1.16 (0.98-1.37) | 0.99 (0.97-1.01) | 4.6% |
| Post-Aus | 1.08 (0.89-1.26) | 1.07 (0.89-1.28) | 1.00 (0.98-1.02) | 0.3% |
| **2013** |  |  |  |  |
| Overall | **1.20 (1.08-1.34)** | **1.19 (1.07-1.34)** | 1.00 (0.99-1.01) | 1.7% |
| Post-Aman | 1.14 (0.94-1.35) | 1.12 (0.93-1.34) | 1.01 (0.995-1.04) | 10.0% |
| Monsoon | 1.21 (0.999-1.39) | **1.22 (1.01-1.41)** | 1.0 (0.98-1.02) | 2.5% |
| Post-Aus | **1.39 (1.07-1.76)** | **1.38 (1.06-1.76)** | 1.00 (0.99-1.02) | 0.4% |
| **2014** |  |  |  |  |
| Overall | 1.07 (0.96-1.18) | 1.07 (0.96-1.19) | 0.99 (0.98-1.01) | 11.2% |
| Post-Aman | 1.11 (0.93-1.35) | 1.12 (0.92-1.36) | 1.0 (0.96-1.03) | 3.0% |
| Monsoon | 0.96 (0.78-1.16) | 0.97 (0.78-1.17) | 0.99 (0.96-1.01) | 29.0% |
| Post-Aus | 1.12 (0.92-1.35) | 1.13 (0.93-1.37) | 0.99 (0.97-1.02) | 7.5% |
|  |  |  |  |  |
|  | **Outcome: Wasting** | | | |
|  | **Total effect** | **Natural Direct Effect** | **Natural Indirect Effect** | **Absolute mediation effect, %** |
|  | **AOR (95% CI^1^)** | **AOR (95% CI^1^)** | **AOR (95% CI^1^)** |  |
| **2012** |  |  |  |  |
| Overall | **1.27 (1.08-1.49)** | **1.27 (1.08-1.49)** | 1.00 (0.99-1.01) | 0.6% |
| Post-Aman | 1.17 (0.82-1.58) | 1.17 (0.85-1.60) | 1.00 (0.98-1.02) | 0.7% |
| Monsoon | 1.07 (0.84-1.42) | 1.06 (0.84-1.43) | 1.01 (0.99-1.04) | 17.7% |
| Post-Aus | **1.88 (1.43-2.59)** | **1.89 (1.43-2.60)** | 0.99 (0.97-1.02) | 1.3% |
| **2013** |  |  |  |  |
| Overall | 1.11 (0.93-1.30) | 1.11 (0.92-1.30) | 1.00 (0.99-1.02) | 1.7% |
| Post-Aman | 0.91 (0.66-1.20) | 0.90 (0.66-1.19) | 1.01 (0.98-1.04) | 9.0% |
| Monsoon | 1.17 (0.92-1.49) | 1.17 (0.91-1.49) | 1.0 (0.97-1.03) | 0.8% |
| Post-Aus | 1.47 (0.95-2.10) | 1.44 (0.94-2.06) | 1.02 (0.99-1.06) | 6.2% |
| **2014** |  |  |  |  |
| Overall | 1.17 (0.96-1.37) | 1.15 (0.97-1.36) | 1.01 (0.99-1.03) | 6.7% |
| Post-Aman | 1.13 (0.82-1.47) | 1.08 (0.77-1.43) | **1.05 (1.003-1.11)** | 39.7% |
| Monsoon | 1.11 (0.85-1.42) | 1.10 (0.84-1.41) | 1.01 (0.99-1.04) | 12.5% |
| Post-Aus | 1.34 (0.999-1.75) | 1.36 (0.99-1.78) | 0.99 (0.95-1.03) | 3.8% |
|  |  |  |  |  |
|  | **Outcome: Underweight** | | | |
|  | **Total effect** | **Natural Direct Effect** | **Natural Indirect Effect** | **Absolute mediation effect, %** |
|  | **AOR (95% CI^1^)** | **AOR (95% CI^1^)** | **AOR (95% CI^1^)** |  |
| **2012** |  |  |  |  |
| Overall | **1.22 (1.10-1.37)** | **1.21 (1.08-1.35)** | **1.01 (1.0003-1.02)** | 5.4% |
| Post-Aman | 1.12 (0.92-1.38) | 1.10 (0.91-1.35) | **1.01 (1.004-1.04)** | 13.7% |
| Monsoon | **1.30 (1.07-1.55)** | **1.29 (1.04-1.54)** | 1.01 (0.996-1.04) | 5.3% |
| Post-Aus | **1.33 (1.09-1.59)** | **1.32 (1.08-1.57)** | 1.01 (0.99-1.03) | 2.0% |
| **2013** |  |  |  |  |
| Overall | **1.17 (1.03-1.30)** | **1.15 (1.02-1.28)** | **1.01 (1.004-1.03)** | 9.9% |
| Post-Aman | 1.15 (0.94-1.39) | 1.13 (0.91-1.37) | 1.01 (0.996-1.04) | 11.4% |
| Monsoon | **1.25 (1.02-1.47)** | **1.23 (1.01-1.45)** | 1.01 (0.99-1.03) | 4.3% |
| Post-Aus | 1.10 (0.87-1.49) | 1.07 (0.84-1.46) | **1.03 (1.003-1.06)** | 33.0% |
| **2014** |  |  |  |  |
| Overall | 1.08 (0.97-1.22) | 1.08 (0.97-1.23) | 1.0 (0.98-1.01) | 1.4% |
| Post-Aman | 1.02 (0.84-1.28) | 1.00 (0.82-1.23) | 1.02 (0.99-1.06) | 88.3% |
| Monsoon | 1.11 (0.93-1.38) | 1.10 (0.92-1.41) | 1.002 (0.99-1.03) | 2.2% |
| Post-Aus | 1.19 (0.98-1.47) | 1.20 (0.99-1.49) | 0.99 (0.96-1.01) | 5.8% |

**Note:** AOR= Adjusted odds ratio, CI^1^= Bias-corrected confidence interval, Bold values denote statistically significant (no “0” values in the CI).
